# The effect of discharge destination and primary insurance provider on hospital discharge delays among patients with traumatic brain injury: a multicenter study of 1,543 patients

**DOI:** 10.1186/s13037-019-0227-z

**Published:** 2020-01-06

**Authors:** Melissa Sorensen, Erica Sercy, Kristin Salottolo, Michael Waxman, Thomas A. West, Allen Tanner, David Bar-Or

**Affiliations:** 1 Trauma Services Department, Swedish Medical Center, Englewood, CO USA; 2 Trauma Research Department, Swedish Medical Center, Englewood, CO USA; 3 Trauma Research Department, Medical City Plano, Plano, TX USA; 4 Trauma Research Department, Research Medical Center, Kansas City, MO USA; 5 Trauma Research Department, Penrose Hospital, Colorado Springs, CO USA; 6 Medical/Surgical Intensive Care Unit, Research Medical Center, Kansas City, MO USA; 7 Trauma Services Department, Medical City Plano, Plano, TX USA; 8 Trauma Services Department, Penrose Hospital, Colorado Springs, CO USA; 9 Injury Outcomes Network Research Group, 501 E Hampden Ave, Englewood, CO 80113 USA

**Keywords:** Patient discharge, Traumatic brain injury, Insurance

## Abstract

**Background:**

Hospital length of stay (HLOS) is a commonly used measure of hospital quality and is influenced by clinical and non-clinical factors. To reduce HLOS, it is key to identify factors placing patients at increased risk of lengthy HLOS and discharge delays.

**Methods:**

This was a retrospective cohort study of patients age ≥ 18 admitted to four level 1 trauma centers between 1/1/2015 and 3/31/2018 with traumatic brain injury (TBI). The primary outcome was discharge delay, defined as discharge ≥24 h after case management notes indicated the patient was ready for discharge. The independent variables of interest were primary insurance provider and discharge destination. Chi-square, Fisher exact, and unadjusted and adjusted logistic regression analyses were used to assess associations between discharge delay and the two primary independent variables, as well as other patient demographic and clinical characteristics. Complications developing during the delay period were also examined.

**Results:**

A total of 1543 patients with TBI were included. The median age was 61 years, and the median HLOS was 5 days. Approximately half of patients were discharged home (54%). The most common insurance providers were Medicare (35%) and commercial/private (35%). Two-hundred ten (14%) patients experienced a discharge delay. The median delay period was 3 days, and the most common reasons for delay were insurance authorization (52%) and lack of accepting bed (41%). Compared to being discharged home, patients discharged to a skilled nursing facility (adjusted odds ratio (AOR) = 10.35) or intermediate care facility (AOR = 10.64) had the highest odds of discharge delay. Compared to Medicare patients, uninsured/self-pay patients (AOR = 2.98) and those with Medicaid (AOR = 2.83) or commercial/private insurance (AOR = 2.22) had higher odds of delay. Thirty-two patients (15% of those delayed) experienced at least one complication during the delay, some of which were clinically severe.

**Conclusions:**

A substantial portion of TBI patients in this study experienced discharge delays, and discharge destination and primary insurance provider were significant drivers of these delays. Evaluation of a facility’s quality of care should consider the specific causes of these delays.

## Background

Among the many measures regularly used to assess hospital quality of care is hospital length of stay (HLOS). Hospital length of stay is used in this way by multiple evaluation organizations, including the Trauma Quality Improvement Program [1] and the Agency for Healthcare Research and Quality Healthcare Cost and Utilization Project [2, 3]. Shorter average HLOS is often an important target of quality-improvement measures because of the association between longer HLOS and development of patient complications, [4–6] including mortality and hospital-acquired infection, as well as increased cost to the hospital, [7]. A key first step in intervening on this issue is identifying factors that place patients at increased risk for lengthy HLOS and delays in hospital discharge.

Multiple factors, both clinical and non-clinical, influence HLOS and discharge delays. Among patients with traumatic injury, previous studies have shown an association between higher injury severity and longer HLOS [8, 9]. It has been suggested that non-clinical factors may also have a major role in HLOS among trauma patients [10–12]. One previous study found that clinical factors accounted for only ~ 20% of hospital discharge delays at their facility over a 5-year period, with the remainder of delays being due to in-hospital procedural delays, insurance-related issues, and placement to rehabilitation facilities at discharge [10]. Other studies using data from level 1 trauma centers and the National Trauma Data Bank found that the strongest predictor of lengthy HLOS was discharge destination [7, 12]. These previous findings point to HLOS being determined by factors that are both under a facility’s control to intervene upon (e.g., hospital operational delays) and those that are not (e.g., patient clinical characteristics, discharge destination availability).

Patients with TBI, especially those with moderate or severe TBI, are at increased risk of lengthy HLOS because of their risk of intracranial hemorrhage, neurological deficits following injury, and potential need for neurosurgical procedures [13, 14]. According to the most recently available Centers for Disease Control and Prevention data, there were ~ 2.87 million TBI-related emergency room visits, hospitalizations, and deaths in 2014, [15] and this group comprises a substantial portion of all trauma patients in the United States. This patient population represents a large group that could be targeted in efforts to reduce HLOS in an individual facility or across a hospital system if modifiable factors can be identified that lead to increased HLOS among TBI patients.

The aim of this study was to focus on a specific group of trauma patients, those with TBI, and examine what clinical and non-clinical factors are associated with increased HLOS attributable to discharge delays. Although anecdotal evidence has suggested that discharge delays and delay-period complications are a significant issue among TBI patients at the level 1 trauma centers included in this study, the goals here were to quantify the scale of the issue and examine what factors—demographic, clinical, or administrative—place TBI patients at the highest risk of experiencing discharge delay, potentially offering targets for future interventions.

## Methods

This was a retrospective cohort study on consecutively admitted trauma patients age 18 and older admitted to one of four level 1 trauma centers across three states between 1/1/2015 and 3/31/2018. This study was approved by the Institutional Review Board at each participating center and was granted a waiver of informed consent and HIPAA authorization. Patients were included if they were admitted to one of the participating centers with an ICD-9 or ICD-10 diagnosis code indicating TBI and an Injury Severity Score (ISS) ≥9. Patients were excluded if they had an ICD-9 or ICD-10 diagnosis of a concussion only and had an Abbreviated Injury Scale (AIS) score > 2 in any body region other than the head. These exclusion criteria aimed to focus on TBI more severe than concussion and increase the probability of the TBI being the primary injury. Patients were evaluated for inclusion and exclusion criteria using the trauma registries at each participating facility. Additional detailed patient information, including case management notes, reasons for discharge delay, insurance utilized, and complications experienced during the discharge delay period, were obtained via abstraction from patient electronic medical records.

Demographic and clinical characteristics collected on patients were age, sex, race, and the following comorbidities: history of substance abuse, presence of an advance healthcare directive, bleeding disorder or current anticoagulant use, cardiovascular disorder (congestive heart failure, hypertension, myocardial infarction, peripheral artery disease, coronary artery disease), cirrhosis of the liver, respiratory conditions (asthma, chronic obstructive pulmonary disease), history of stroke, dementia, functionally dependent health status, major psychiatric disorder, and current smoking. Injury and clinical characteristics included admission ISS, admission Glasgow Coma Scale (GCS) score, total HLOS, intensive care unit (ICU) LOS, mechanism of injury (fall, motor vehicle collision, assault, other), discharge disposition (home/home health, rehabilitation facility, skilled nursing facility (SNF), hospice, intermediate care facility (ICF), long-term acute care (LTAC)/nursing home, other, death), and undergoing a neurosurgical intervention (craniotomy, craniectomy, craniostomy, burr hole, surgical elevation of skull fracture, placement of intracranial pressure monitor, ventriculostomy) during hospital stay. Administrative details collected were primary insurance provider (commercial/private, Medicare, Medicaid, Military, uninsured/self-pay, other), utilization of secondary insurance, and secondary insurance provider.

The primary outcome was experiencing a discharge delay, defined here as discharge ≥24 h after case management notes indicated the patient was medically ready for discharge. The primary independent variables of interest were discharge disposition and primary insurance provider; however, all demographic, clinical, and administrative factors were also examined for associations with discharge delay. Chi-square or Fisher exact tests were used to examine differences in demographic, clinical, and administrative characteristics between patients who experienced delay and those who did not. Unadjusted and adjusted logistic regression analyses were used to further investigate associations between demographic, clinical, and administrative characteristics and experiencing a discharge delay. In the adjusted model, the two independent variables of interest, discharge disposition and primary insurance provider, and all variables that showed significance in unadjusted models were made available for inclusion in the final model. Stepwise selection with entry and exit criteria of α = 0.05 was used to determine the variables remaining in the final adjusted model.

Further analyses were used to describe the discharge delay periods in more detail. The median days between admission and start of delay and the median total delay days were calculated, and the reasons for the discharge delays were described. In addition, complications that developed during the discharge delay period were collected, as well as the median hours between the beginning of the delay period and development of the first post-delay complication. All statistical analyses were conducted in SAS version 9.4.

## Results

This study included a total of 1543 patients with TBI admitted to the four participating centers over the study period. The median age of the patient population was 61 years, 60% were male, and the majority were white (79%) (Table 1). The most common comorbidities were a cardiovascular condition (45%), current smoking (20%), substance abuse (17%), and diabetes (15%). The most common cause of injury was a fall (60%), followed by a motor vehicle collision (20%). The median ISS was 14, and the median GCS score was 15, indicating only minor cognitive impairment among most of the patient population. The median HLOS was 5 days, and the median ICU LOS among those patients with ICU stays was 2 days. One-hundred eighty-six patients (12%) had a neurosurgical intervention during their hospital stay.

**Table 1:** Differences in clinical and administrative factors in patients with and without discharge delays

	Overall study population	Delayed	Not Delayed	P
	*n* = 1543	*n* = 210 (13.6%)	*n* = 1333 (86.4%)	
*Delay Details*				
Days from admission to delay start, median (IQR), range	–	6 (3–12)	–	
Delay days, median (IQR)	–	3 (2–6)	–	
Reason for delay				
Insurance	–	109 (51.9%)	–	
Accepting bed	–	85 (40.5%)	–	
Patient	–	72 (34.3%)	–	
Provider	–	12 (5.7%)	–	
Procedure	–	9 (4.3%)	–	
Test	–	9 (4.3%)	–	
HLOS days, median (IQR)	5 (3–9)	10 (7–19)	4 (2–7)	**< 0.01**
ICU LOS days, median (IQR)	2 (1–5)	5 (3–10)	2 (1–4)	**< 0.01**
*Patient Demographics*				
Age, median (IQR)	61 (40–78)	63 (46–79)	61 (39–77)	0.27
Sex				0.61
Male	923 (59.8%)	129 (61.4%)	794 (59.6%)	
Female	620 (40.2%)	81 (38.6%)	539 (40.4%)	
Race				0.64
White	1187 (78.9%)	164 (81.6%)	1023 (78.5%)	
Black	151 (10.0%)	20 (10.0%)	131 (10.1%)	
Hispanic	23 (1.5%)	2 (1.0%)	21 (1.6%)	
Other	143 (9.5%)	15 (7.5%)	128 (9.8%)	
*Clinical Characteristics*				
ISS, median (IQR)	14 (10–21)	17 (11–25)	14 (10–20)	**< 0.01**
GCS, median (IQR)	15 (14–15)	14 (8–15)	15 (14–15)	**< 0.01**
Comorbidities				
Cardiovascular	692 (44.9%)	98 (46.7%)	594 (44.6%)	0.57
Smoker	309 (20.0%)	38 (18.1%)	271 (20.3%)	0.45
Substance abuse	263 (17.0%)	41 (19.5%)	222 (16.7%)	0.30
Diabetes	236 (15.3%)	34 (16.2%)	202 (15.2%)	0.70
Bleeding disorder	166 (10.8%)	22 (10.5%)	144 (10.8%)	0.89
Dementia	137 (8.9%)	23 (11.0%)	114 (8.6%)	0.26
Psychiatric	121 (7.8%)	33 (15.7%)	88 (6.6%)	**< 0.01**
Respiratory	108 (7.0%)	18 (8.6%)	90 (6.8%)	0.34
Previous stroke	103 (6.7%)	17 (8.1%)	86 (6.5%)	0.38
Functionally dependent health status	101 (6.6%)	12 (5.7%)	89 (6.7%)	0.60
Advanced directive	51 (3.3%)	9 (4.3%)	42 (3.2%)	0.39
Cirrhosis	22 (1.4%)	7 (3.3%)	15 (1.1%)	**0.02**
Mechanism of injury				**0.01**
Fall	924 (59.9%)	117 (55.7%)	807 (60.5%)	
Motor vehicle collision	306 (19.8%)	59 (28.1%)	247 (18.5%)	
Assault	87 (5.6%)	9 (4.3%)	78 (5.9%)	
Other	226 (14.7%)	25 (11.9%)	201 (15.1%)	
Neurosurgical intervention	186 (12.1%)	40 (19.1%)	146 (11.0%)	**< 0.01**
Neurosurgical intervention prior to delay	–	35 (16.7%)	–	**–**
*Administrative*				
Discharge disposition				**< 0.01**
Home/home health	826 (53.5%)	43 (20.5%)	783 (58.7%)	
Rehabilitation facility	284 (18.4%)	78 (37.1%)	206 (15.5%)	
Skilled nursing facility	157 (10.2%)	44 (21.0%)	113 (8.5%)	
Hospice	78 (5.1%)	5 (2.4%)	73 (5.5%)	
Intermediate care facility	68 (4.4%)	18 (8.6%)	50 (3.8%)	
Long-term acute care/nursing home	51 (3.3%)	14 (6.7%)	37 (2.8%)	
Other	46 (3.0%)	4 (1.9%)	44 (3.3%)	
Death	31 (2.0%)	4 (1.9%)	27 (2.0%)	
Primary insurance				0.32
Medicare	539 (35.0%)	62 (29.7%)	477 (35.9%)	
Commercial/private	534 (34.7%)	81 (38.8%)	453 (34.1%)	
Uninsured/self-pay	204 (13.3%)	28 (13.4%)	176 (13.2%)	
Medicaid	148 (9.6%)	25 (12.0%)	123 (9.3%)	
Other	83 (5.4%)	11 (5.3%)	72 (5.4%)	
Military	31 (2.0%)	2 (1.0%)	29 (2.2%)	
Secondary insurance utilized	207 (13.4%)	40 (19.1%)	167 (12.5%)	**< 0.01**
Secondary insurance				0.43
Medicare	47 (22.7%)	9 (22.5%)	38 (22.8%)	
Commercial/private	70 (33.8%)	9 (22.5%)	61 (36.5%)	
Self-pay	25 (12.1%)	5 (12.5%)	20 (12.0%)	
Medicaid	33 (15.9%)	10 (25.0%)	23 (13.8%)	
Other	19 (9.2%)	4 (10.0%)	15 (9.0%)	
Military	13 (6.3%)	3 (7.5%)	10 (6.0%)	

*IQR* interquartile range; *HLOS* hospital length of stay; *LOS* length of stay; *ICU* intensive care unit; *ISS* Injury Severity Score; *GCS* Glasgow Coma Scale. Bold indicates statistical significance at a threshold of *P* < 0.05. Unless otherwise indicated, results are shown as n (%)

The most common discharge destination was home or home health (54%), followed by a rehabilitation facility (18%), and a SNF (10%). The most commonly utilized primary insurance providers were Medicare (35%) and commercial/private insurance (35%). Two-hundred seven patients (13%) utilized a secondary form of insurance, and the most common secondary insurance provider was commercial/private insurance (34% of those using a secondary insurance provider).

Of the 1543 total TBI patients, 210 (14%) experienced a discharge delay. The median time between admission and delay start was 6 days, and the median delay period was 3 days, meaning that patients remained in the hospital for approximately 3 days after case management notes indicated they ready for discharge (Table 1). The most common reason for the delay was insurance processing or authorization (52% of patients with delay), followed by lack of an accepting bed at the receiving facility (41% of patients with delay) and patient-related reasons (e.g., language barrier, coordination with family members; 34% of all delayed patients). Total HLOS and ICU LOS were both significantly longer in delayed patients (HLOS: 10 days vs 4 days for non-delayed patients, *P* < 0.01; ICU LOS: 5 days vs 2 days, *P* < 0.01).

Clinical factors univariately associated with delay included presence of a psychiatric comorbidity (16% vs 7% of non-delayed patients, *P* < 0.01) and cirrhosis of the liver (3% vs 1%, *P* = 0.02) (Table 1), a higher median ISS (17 vs 14, *P* < 0.01) and motor vehicle collision as the injury cause (28% vs 19%, *P* < 0.01). The median (IQR) GCS score for delayed patients was 14 (8–15) compared to 15 (14–15) for non-delayed patients (*P* < 0.01), indicating that delayed patients had a larger range of GCS scores and were more likely to have cognitive impairment than non-delayed patients. Delayed patients were more likely to have undergone a neurosurgical intervention during their hospital stay (19% vs 11%, *P* < 0.01). Of the total delayed patients who underwent neurosurgical intervention (*n* = 40), 35 of these patients (88%) had the neurosurgical intervention prior to the start of the delay. Administrative factors univariately associated with delay were hospital discharge to a facility other than home (*P* < 0.01) and utilization of a secondary form of insurance (19% vs 13%, *P* < 0.01). Primary insurance status was not associated with discharge delay prior to adjustment.

In adjusted logistic regression analyses, both clinical and administrative features were significantly associated with experiencing discharge delay (Table 2). Both discharge destination and primary insurance provider were significantly associated with discharge delay. Patients discharged to a SNF (OR = 10.35, 95% CI 6.06–17.96) or ICF (OR = 10.64, 95% CI 5.27–21.46) were the most likely to have a delay compared to those discharged home. Patients utilizing commercial/private insurance (OR = 2.22, 95% CI 1.46–3.38), Medicaid (OR = 2.83, 95% CI 1.52–5.25), or another insurance type (OR = 2.32, 95% CI 1.01–5.30) as their primary insurance provider and those who were self-pay or uninsured (OR = 2.98, 95% CI 1.62–5.47) were more likely to have a delay than those utilizing Medicare. Patients with cirrhosis (OR = 3.93, 95% CI 1.39–11.13) and those with psychiatric comorbidities (OR = 2.99, 95% CI 1.80–4.96) were significantly more likely to experience discharge delay. In addition, a lower GCS score, indicating more severe cognitive impairment, was significantly associated with discharge delay (OR = 0.95, 95% CI 0.91–0.99).

**Table 2:** Associations of clinical and administrative factors with experiencing discharge delay

	Unadjusted	Adjusted
	OR (95% CI)	OR (95% CI)
*Patient Demographics*		
Age	1.00 (0.99–1.01)	
Sex		
Male	Ref	
Female	0.93 (0.69–1.25)	
Race		
White	Ref	
Black	0.95 (0.58–1.57)	
Hispanic	0.59 (0.14–2.56)	
Other	0.73 (0.42–1.28)	
*Clinical Characteristics*		
ISS	**1.04 (1.02–1.06)**	
GCS	**0.90 (0.87–0.93)**	**0.95 (0.91–0.99)**
Comorbidities		
Substance abuse	1.21 (0.84–1.76)	
Advanced directive	1.38 (0.66–2.87)	
Bleeding disorder	0.97 (0.60–1.55)	
Cardiovascular	1.09 (0.81–1.46)	
Cirrhosis	**3.03 (1.22–7.52)**	**3.93 (1.39–11.13)**
Respiratory	1.30 (0.76–2.20)	
Previous stroke	1.28 (0.74–2.20)	
Dementia	1.32 (0.82–2.11)	
Functionally dependent health status	0.85 (0.46–1.58)	
Diabetes	1.08 (0.73–1.61)	
Psychiatric	**2.64 (1.72–4.06)**	**2.99 (1.80–4.96)**
Smoker	0.87 (0.60–1.26)	
Mechanism of injury		
Fall	Ref	
Motor vehicle collision	**1.65 (1.17–2.32)**	
Assault	0.80 (0.39–1.63)	
Other	0.86 (0.54–1.36)	
Discharge disposition		
Home/home health	Ref	Ref
Rehabilitation facility	**6.90 (4.61–10.31)**	**8.12 (5.12–12.89)**
Skilled nursing facility	**7.09 (4.46–11.28)**	**10.35 (6.06–17.96)**
Hospice	1.25 (0.48–3.25)	0.82 (0.23–2.91)
Intermediate care facility	**6.56 (3.53–12.19)**	**10.64 (5.27–21.46)**
Long-term acute care/nursing home	**6.89 (3.47–13.70)**	**6.16 (2.77–13.70)**
Other	1.66 (0.57–4.82)	1.23 (0.41–3.72)
Death	2.70 (0.90–8.06)	2.24 (0.68–7.31)
Neurosurgical intervention prior to delay	**2.44 (1.61–3.70)**	**1.84 (1.12–3.01)**
*Insurance*		
Primary insurance		
Medicare	Ref	Ref
Commercial/private	1.38 (0.97–1.96)	**2.22 (1.46–3.38)**
Uninsured/self-pay	1.22 (0.76–1.98)	**2.98 (1.62–5.47)**
Medicaid	1.56 (0.94–2.59)	**2.83 (1.52–5.25)**
Other	1.18 (0.59–2.34)	**2.32 (1.01–5.30)**
Military	0.53 (0.12–2.28)	0.72 (0.14–3.51)
Secondary insurance utilized	**1.64 (1.12–2.40)**	
Secondary insurance		
Medicare	Ref	
Commercial/private	0.62 (0.23–1.71)	
Self-pay	1.06 (0.31–3.58)	
Medicaid	1.84 (0.65–5.19)	
Other	1.13 (0.30–4.22)	
Military	1.27 (0.29–5.57)	

*OR* odds ratio; *95% CI* 95% confidence interval; *ISS* Injury Severity Score; *GCS* Glasgow Coma Scale. Ref indicates the reference group in the logistic models. Bold indicates statistical significance at a threshold of *P* < 0.05

Thirty-two patients (15% of delayed patients) experienced at least one complication during their delay period, and 16 patients (8%) experienced at least two complications (Table 3). The median time between the beginning of the delay period and development of the first complication was 71 h. The most common complications were altered mental status (increasing anxiety or anxiety attack, delirium, increasing distress; 5%), altered blood labs (hypoxia, anemia, hyponatremia, leukocytosis; 4%), and urinary tract infection (3%). Among the most clinically severe complications during the delay period were development of a brain abscess (*n* = 1), enlarged brain ventricles (*n* = 1), deep vein thrombosis (*n* = 1), and new-onset intracranial hemorrhage (*n* = 1). Four patients (2% of delayed patients) died during their discharge delay period.

**Table 3:** Delay-period complications

Complication Details	Total *n* = 210
Experienced ≥1 delay-period complication	32 (15.2%)
Hours from delay start to delay-period complication, median (IQR)	71 (23–153)
Complication during delay	
Altered mental status^a^	11 (5.2%)
Altered blood labs^b^	8 (3.8%)
Urinary tract infection	7 (3.3%)
Neurological complications^c^	4 (1.9%)
Lung complications^d^	4 (1.9%)
Pneumonia	3 (1.4%)
Rash/allergic reaction	3 (1.4%)
Thrombocytosis	3 (1.4%)
Superficial vein thrombosis^e^	2 (1.0%)
Deep vein thrombosis	1 (0.5%)
New-onset intracranial hemorrhage	1 (0.5%)
Death during delay period	4 (1.9%)
Number of complications during delay	
0	178 (84.8%)
1	16 (7.6%)
≥ 2	16 (7.6%)

*IQR* interquartile range

^a^Altered mental status included increasing anxiety or anxiety attack, delirium, and increasing distress. ^b^Altered blood labs included hypoxia, anemia, hyponatremia, and leukocytosis. ^c^Neurological complications included brain abscess, dysphasia, enlarged brain ventricles, and diplopia. ^d^Lung complications included bilateral pleural effusions, atelectasis, retained left hemothorax, and bronchial herpes simplex type 1. ^e^Veins affected were the basilic vein and the cephalic vein. Unless otherwise indicated, results are shown as n (%)

## Discussion

Hospital length of stay is often used to assess the quality of care provided at a facility, as lengthy HLOS and discharge delays have been associated with in-hospital complications, patient mortality, and increased cost to the hospital. This issue is especially pertinent among patients with TBI, as they comprise a substantial portion of the total trauma population and are already at increased risk for longer HLOS and complications because of the severity and nature of their injuries. This study found that among patients with TBI admitted to four level 1 trauma centers over ~ 3 years, a substantial portion (14%) experienced delays in hospital discharge. The factors most strongly associated with experiencing delay were discharge destination, primary insurance provider, and certain patient comorbidities.

Previous studies have examined the reasons behind lengthy HLOS and discharge delays among trauma patients, with the aim of identifying areas for potential intervention and procedural change. Some of these reasons identified previously, such as hospital operational delays, [10] represent good targets for quality-improvement measures. Additionally, clinical factors associated with increased HLOS, such as injury severity [8, 9], lower GCS, and the presence of certain comorbidities, as found here, may help identify specific patient groups at higher risk for longer HLOS and discharge delays. However, consistent with previous studies that found discharge destination to be a major factor influencing HLOS, [7, 12] we found that two external administrative factors were the primary drivers of hospital discharge delays among TBI patients in our study: primary insurance provider and discharge destination.

Compared to patients discharged home, those being discharged to a SNF or ICF had the highest odds of experiencing discharge delay. Patients utilizing Medicare as their primary insurance provider had the lowest odds of delay, and patients utilizing commercial/private insurance or Medicaid and patients who were uninsured or self-pay had increasing odds of delay. Consistent with our findings, a previous study examining delays in placement to nursing homes found that utilizing Medicaid as a primary insurance provider was significantly associated with delays in discharge and placement [16]. Also in accordance with our results here, a previous study in stroke patients found that private/commercial insurance providers frequently require pre-certification before discharge to a SNF or inpatient rehabilitation facility, resulting in substantial discharge delays for patients with this type of primary insurance [17]. Our finding that the most common causes of delay were insurance processing and authorization and lack of available beds at the discharge destination, in conjunction with the findings of previous studies, imply that a complex network of approval and communication between insurance providers and discharge locations exists that has the potential to result in lengthy HLOS and discharge delays.

This study also found that among patients who experienced discharge delays, 15% had at least one complication develop during the delay period, consistent with previous studies linking increased HLOS with increased complications [4–6]. The most common complication here was altered mental status, although some of the less frequent complications were more clinically severe, such as brain abscess, enlarged brain ventricles, deep vein thrombosis, and new-onset intracranial hemorrhage. Importantly, four patients, or 2% of all delayed patients, died during their delay period. It is unknown whether the increased HLOS was the cause of many of these complications, which may have developed regardless of the patient’s location. However, it is possible that complications such as pneumonia or urinary tract infection may plausibly be associated with a lengthy hospital stay.

A potential limitation of this study was an inability to capture the many detailed and varying insurance provider policies and practices that affect hospital discharge timeliness, in addition to those examined here. For example, this study did not consider the day of the week on which the patient was ready for discharge; discharge readiness on a Friday afternoon or prior to a holiday may result in discharge delays because of insurance company closure or personnel shortages. It may be worth parsing insurance-related delays further than what was done here to enable specifically targeted interventions to eliminate such delays. It may additionally be useful to examine insurance reimbursement policies during delay periods, as well as what increased costs the insurance provider, hospital, and patient may incur during these delays.

An additional potential limitation of this study was the definition of discharge delay used: discharge ≥24 h after case management notes indicated the patient was ready. This may have been too stringent a cutoff, although it is likely that the appropriate definition of delay depends on typical practices at individual facilities. This study was also not able to assess the cause or directionality of the relationship between complications and discharge delays. We cannot say that longer HLOS caused the complications observed here, although we can report that a significant association existed among our patient population. In addition, it is likely that complications that developed during the delay period resulted in further additional delays, although we were not able to assess this here. The findings of this study are directly applicable to one subset of hospitalized patients only: those with TBI. Additionally, insurance reimbursement policies, availability of discharge destination beds, and other factors affecting discharge timeliness may differ by region, state, and rurality of the facility and may not directly reflect the conditions present at the facilities included here. However, despite these differences, it is likely that other groups of trauma patients experience similar issues with insurance and discharge destination delays. The large sample size and inclusion of four level 1 trauma centers across three states ensured that our study had sufficient power to examine differences in characteristics between delayed and non-delayed patients and conduct adjusted analyses to look for independent associations with the outcome.

## Conclusions

It is worth exploring whether insurance processing delays and communication with the discharge destination can be intervened upon at the acute care hospital, prior to discharge. Perhaps hospital resources may be devoted to additional staff that facilitate smoother and more timely processing of discharge orders and insurance claims, acting as a mediator between insurance providers and discharge destinations. Although it appears that there may be no available action at the discharging hospital in response to a lack of available beds at the receiving facility, an awareness of the shortage, as well as the associated medical and financial consequences, at a systems or regional level may help shape facility planning on a larger scale. Lengthy HLOS and discharge delays are a multi-faceted issue, and it is worth considering the specific causes of these delays and what solutions may be implemented to address them when evaluating the quality of care at an individual facility.

## Data Availability

The datasets generated and/or analyzed during the current study are not publicly available because they contain patient data but are available from the corresponding author on reasonable request.

## Abbreviations

AIS: Abbreviated Injury Scale
AOR: Adjusted odds ratio
CI: Confidence interval
GCS: Glasgow Coma Scale
HLOS: Hospital length of stay
ICF: Intermediate care facility
ICU: Intensive care unit
IQR: Interquartile range
ISS: Injury Severity Score
LOS: Length of stay
LTAC: Long-term acute care
OR: Odds ratio
SNF: Skilled nursing facility
TBI: Traumatic brain injury
